# Communication of medical errors in a simulated clinical scenario. Experience with a pediatric residency group

**DOI:** 10.1590/1984-0462/2024/42/2022109

**Published:** 2023-07-10

**Authors:** María Pico, Ximena Prado, Gonzalo Germán Guiñazú, Sofía Diana Menéndez, Julia Dvorkin, María Victoria López, Carolina Pascual, Christian Elias Costa, Diego Enríquez

**Affiliations:** b Simulación Médica Roemmers, Buenos Aires, Argentina.

**Keywords:** Medical error, High-fidelity simulation, Communication, Medical education, Erro médico, Simulação de alta fidelidade, Comunicação, Educação médica

## Abstract

**Objective:**

To determine the performance of groups of pediatric residents from a Buenos Aires hospital, in terms of correct recognition and communication of a medical error (ME), in a high-fidelity simulation scenario. To describe the reactions and communication attempts following the ME and the self-perception by the trainees before and after a debriefing.

**Methods:**

Quasi-experimental uncontrolled study conducted in a simulation center. First- and third-year pediatric residents participated. We designed a simulation case in which an ME occurred and the patient deteriorated. During the simulation, participants had to provide information on communicating the ME to the patient’s father. We assessed communication performance and, additionally, participants completed a self-perception survey about ME management before and after a debriefing.

**Results:**

Eleven groups of residents participated. Ten (90.9%) identified the ME correctly, but only 27.3% (n=3) of them reported that a ME had occurred. None of the groups told the father they were going to give him important news concerning his son’s health. All 18 residents who actively participated in this communication completed the self-perception survey, with an average score before and after debriefing of 5.00 and 5.05 (out of 10) (p=0.88).

**Conclusions:**

We observed a high number of groups that recognized the presence of a ME, but the communication action was substantially low. Communication skills were insufficient and residents’ self-perception of error management was regular and not modified by the debriefing.

## RESUMO

**Objetivo:**

Determinar o desempenho de grupos de residentes pediátricos de um hospital de Buenos Aires, em termos de reconhecimento e comunicação correta de um erro médico (EM),em cenário de simulação. Descrever as reações e tentativas de comunicação após o EM e a autopercepção pelos estagiários antes e depois de um questionário.

**Métodos:**

Estudo quase experimental não controlado realizado em centro de simulação. Participaram residentes pediátricos do primeiro e terceiro anos. Concebeu-se um caso de simulação em que ocorreu um EM com deterioração de um paciente. Durante a simulação, os participantes tiveram que fornecer informações relacionadas à comunicação do EM ao pai do paciente. Avaliou-se o desempenho da comunicação e, adicionalmente, os participantes completaram um inquérito de autopercepção sobre a gestão da EM, antes e depois de um questionário.

**Resultados:**

Onze grupos de residentes participaram. Dez (90,9%) identificaram corretamente o EM, mas apenas 27,3% (n=3) deles comunicaram que havia ocorrido o EM. Nenhum dos grupos disse ao pai que iria dar notícias importantes sobre a saúde do seu filho. Todos os 18 residentes que participaram ativamente da comunicação completaram o questionário de autopercepção com uma pontuação média antes e depois do questionário de 5,00 e 5,05 (máximo: 10 pontos) (p=0,88).

**Conclusões:**

Observamos elevado número de grupos que reconheceram a presença de um EM, mas a ação de comunicação foi rara. A capacidade de comunicação foi insuficiente e a autopercepção da gestão de erros por parte dos residentes foi regular, não sendo modificada pelo *debriefing*.

## INTRODUCTION

Since the publication of *To Err is Human* ^1^ in 2000 by the Institute of Medicine of the United States, which reported preventable adverse events as one of the main causes of death in the American health system, the aspects related to patient quality of care and safety began to be a priority for health systems. When a medical error (ME) occurs, effective patient-physician communication is essential to achieve a satisfactory outcome for both the patient and their family, as well as for the physician and/or the team involved. Medical ethics guidelines and handbooks highlight the responsibility of healthcare providers in ME communication.^2,3^ However, there are often differences between the patient’s expectations in terms of how the ME should be communicated and the information they actually receive.^4^ A survey conducted in 2005 in five countries with more than 20,000 participants showed that, although patients appreciate the communication and talk about the errors, 60–80% of the surveyed subjects perceived that the healthcare providers involved did not communicate their errors adequately.^5^ Pediatric studies describe that virtually all parents would expect to be informed if a ME occurs during their child’s care.^6^ These findings show that reporting an error is a very challenging task for healthcare providers, where emotions and personal and environmental demands may lead to unethical decisions.^7,8^ Moreover, ME emotionally affect the healthcare provider, who is usually defined as the second victim.^9^ In this context, healthcare providers are often reluctant to some extent to communicate errors, thus turning to concealment and avoidance.^10^ Within this scenario, it is crucial that different tools are available to them so they can develop and improve their knowledge, attitudes and skills for communicating ME in everyday practice.^11^

Educating teams responsible for the training of healthcare providers play a significant, yet challenging, role in teaching adequate error prevention and communication. Residents are expected to establish effective communication with patients and families, but there is often little training for them to acquire the required skills. In this context, simulation-based medical education has shown to be effective in safely providing both the knowledge and the skills that healthcare providers should acquire,^12^ encouraging them to develop interpersonal and communication skills.^13^ Previous experiences on the use of simulated error communication training^14,15^ have provided quantitative proof for the presence or absence of such abilities and helped to identify opportunities for communication improvement.

The primary objective of this study was to assess the performance of groups of pediatric residents from a Buenos Aires hospital in a high-fidelity simulation scenario, in terms of correct recognition and communication of a ME. The secondary objective was to describe the reactions and communication attempts following the ME and the self-perception by resident physicians before and after a thought-provoking, structured and object-based conversation aimed at learning, keeping or improving professional practice (debriefing).

## METHOD

We conducted a quasi-experimental non-controlled study including ten sessions in the high-fidelity simulation medical center SIMMER. All residents who participated in the simulation training days were included. Study participants were first- and third-year residents from a tertiary care children’s hospital in Buenos Aires, who were part of the study during the months of January and February 2020. Residents from other years were not included because the pediatric program offers high-fidelity simulation training specifically for these years due to availability.

We designed a high-fidelity, highly realistic simulation case, where the patient presented with decompensation secondary to a ME in the context of a condition for which he was receiving care. The case required, as a first measure, recognizing the error situation and considering whether or not to report the ME to the patient’s father, who was asking for more information. Either one or two residents could carry out the communication with the patient’s father. The setting included providing staff from the simulation center with pre-established scripts for them to play characters such as the nurse, who failed to administer the correct drug dose, and the patient’s father. All scenarios were filmed for educational purposes upon consent by the participants. The case involved a teenage patient with seizures at the Emergency Room. As previously established, during the simulation, one of the facilitators in charge of the simulation made a ME, administering ten times the prescribed dose of benzodiazepine. Because of this error, the patient developed a respiratory arrest, which required advanced resuscitation maneuvers. Although, at the start of the case, the patient was accompanied by his father (role played by the same actor in all sessions), the father left the setting spontaneously and did not witness the moment of the ME. The resident physicians were then supposed to identify the error and consider whether or not to communicate what had happened to the patient’s father, who was now actively asking how his son had suddenly ended up in such condition. We prepared a script for the person playing the father, considering the potential scenarios of interaction with the residents and their answers, and participants were always addressed respectfully and without violence.

In order to assess the communication performance of the 11 participating groups, we prepared an assessment form based on the García Díaz’s bad news communication protocol.^15^ The assessment form included 13 yes/no questions regarding ME recognition and communication, ME contextualization and narrative, emotion management, and empathy (Table 1). Once the sessions were completed, we analyzed the recorded scenes to objectively assess communication. Three researchers carried out the assessment independently. For the mismatching yes/no answers, they carried out a second round of assessments in order to reach a consensus on the outcome, reporting the number of affirmative answers for each of the questions. Although formal education in bad news communication is not available in our setting, the researchers had experience communicating and teaching in a simulation scenario.^17,18,19^

**Table 1.** ME Communication Performance Assessment Form.^15^

	Yes	No
Error		
1. Recognizes the error.		
2. Recognizes the error with the aid of a facilitator.		
3. Communicates the error.		
Context		
4. Keeps eye contact when speaking.		
5. Keeps eye contact when listening.		
6. Places him/herself level with the patient’s father (sitting-sitting/standing-standing).		
Narrative		
7. Informs the family that (s)he will give some important news.		
8. Uses plain and adequate language with the family member.		
9. Lets the family member speak without interruptions. Makes pauses.		
10. Avoids blaming.		
11. Apologizes.		
Emotions and empathy		
12. Avoids assuring the family member that there will be a good outcome or that no damage was done.		
13. Handles the father’s emotional reaction adequately.		

Observer: _______________________ Date: _______________________ Comments:________________________________________________________________________________________________________________

The secondary objective included the evaluation of self-perception regarding error communication. All the residents who communicated the error were included in this analysis. Upon completion of the simulation scenario, the participants assessed their own error communication performance based on a Likert-type scale from 0 to 10 (Table 2). Additionally, they answered whether or not this was the first time they had reported a ME and if they had any prior related training. Afterwards, some of the simulation trainers led a debriefing to consolidate the perceptions and lessons learned through the simulation (delta-plus debriefing model).^20^ Following the debriefing, the participants made a new self-assessment using the same scale. After collecting the data, we calculated the mean pre- and post-debriefing error management scores and tested the difference between both scores for significance (paired samples Wilcoxon test). For the statistical analysis, we used STATA version 14 statistical package (StataCorp. 2015. Stata Statistical Software: Release 14. College Station, TX: StataCorp LP). Prior to the study, we requested approval from the hospital’s Teaching Committee and Ethics Committee and ensured the confidentiality of all collected data.

**Table 2.** Self-perception assessment sheet.

Medical error survey — Before debriefing
Date:
Name:
Year of residency
Is this the first time you’ve had to communicate an error as a member of the health staff?
Based on your self-perception, please assess your performance when communicating the medical error on a scale from 0 to 10
If you wish, you may leave a comment:
**Medical error survey — After debriefing**
Date:
Name:
Year of residency
Based on your self-perception and considering the analysis performed on the debriefing, please reassess your performance when communicating the medical error on a scale from 0 to 10
If your self-perception has changed with respect to your previous answer, please explain why you made the change:

## RESULTS

The Ricardo Gutiérrez Children Hospital’s pediatric residency has 160 residents and takes place in the city of Buenos Aires. Forty-six first- and third-year residents attended the simulation sessions and were randomized to 11 groups of three to five participants each. All groups had at least two third-year residents, and their main characteristics are described in Table 3.

**Table 3.** Participant’s background (n=46).

Year of residency	Mean age in years (SD)	Female (%)	Male (%)	Basic residency (%)	Coordinated residency* (%)	Total
1	26.7 (0.8)	7 (70)	3 (30)	8 (80)	2 (20)	10
3	28.8 (0.8)	30 (83.3)	6 (16.7)	32 (88.9)	4 (11.1)	36

*Pediatric Intensive Care Unit or coordinated Neonatology residency program of the autonomous city of Buenos Aires.

Evaluation of the communicative performance of the groups with the use of the observation form yielded the following results: ten groups (90.9%) recognized the error correctly and one (9.1%) required a facilitator to guide the team answers. In all cases, the patient’s father actively asked the participants why his son had suddenly experienced a serious condition, demanding explanations from the intervening group. Only three groups (27.3%) informed the father that a ME had occurred, but none of them told the father that they were going to give him important news concerning his son’s health. During the conversations with the father, nine groups (81.8%) kept eye contact when speaking, and only five kept it while listening (45.5%). Nine groups (81.8%) placed themselves at the same level as the father during the communication process. Six (54.5%) used plain and adequate language and let the father speak without interruptions. Ten groups avoided blaming (90.9%), but none of them apologized for the event. Seven (63.6%) avoided ensuring the family member that the outcome would be favorable, and ten (90.9%) avoided saying that no damage had been done. Out of all the groups, six (54.5%) handled the father’s emotional reaction adequately.

When the simulation scenario was completed, all the residents who had actively participated in the conversation with the patient’s father filled in the self-perception survey (n=18) (Table 2). Of that group, seven (38.9%) participants answered that this was the first time they were facing the situation of communicating a ME, and 16 (88.8%) reported having little or no training in medical error communication. The average self-perception score for communicative performance (on a scale of 0 to 10) was 5. Upon grading themselves again after the debriefing process, the score was 5.05, a non-significant statistical difference (p=0.97).

## DISCUSSION

In the present study, we were able to observe a high percentage of regular performance in ME communication, which coincided with the residents’ self-perception. It is worth mentioning that the residents were not informed about the content of the simulation scenario, i.e., they did not know that they would have to actively participate in communicating an EM. More than half of the participants had already faced the situation of communicating an error, while the majority reported having little or no related training. The average self-perception score was low, which confirms the need to reinforce training in ME communication. Currently in our setting, ME communication is not part of the residency training program, and this type of experience could be useful if applied systematically as part of the professional training process.

Remarkably, less than one third of the groups communicated the error to the patient’s father. This low rate of error communication agrees with that reported in the literature.^21,22^ In this respect, Loren *et al.* published, in 2008, a survey including more than 200 pediatricians in the United States and aimed at learning more about ME communication.^7^ They found out that only 53% of healthcare providers disclosed the ME in its entirety, while only 26% offered an explicit apology. This is not so far from our findings, with 27.3% (n=3) of participants not effectively disclosing the ME, and may be due to cultural factors affecting medical training and fear of the consequences entailed by their active or passive ME, particularly because of the expected patient or family reaction.^23^ Nevertheless, it is interesting to note that many lawsuits are the result of a poor conveyance of information or miscommunication with the patient and their family.^24,25^ A survey of more than 1,200 patients in the United States revealed that only 30% of those who thought they had undergone a ME during their care had received information from the treating healthcare provider about the occurrence of the ME.^26^

Error communication may have a negative impact on the working teams. To face this difficulty, conducting debriefing processes after critical events may help reduce this impact. However, debriefings may be hard to implement in everyday practice due to lack of time, lack of experience in performing this kind of exercise by the health staff, or lack of institutional support. In this study, the score did not vary significantly before and after the debriefing, probably because the baseline self-perception was low. The communicative aspect is classified into the so-called “soft skills”, a common aspect of which is the need for practice, which may account for the scarce score modification after the debriefing session. Importantly, training in communication skills should not overlook the need to improve processes aimed at reducing the rate of errors in clinical practice, such as the double check in medication prescription. Although this is challenging in the emergency setting, its implementation may reduce the rate of errors.

Among the possible strategies for developing communication skills in training health staff, high-fidelity simulation is certainly a valuable practice. The use of simulation settings allows training and “live” performance assessment, and the high fidelity or realism of scenarios helps creating situations similar to those which might occur during everyday clinical practice. It is worth noting that Pediatrics is a specialty where the development of communication skills is particularly relevant because of the need to interact with the patient and their parents and/or carers.^22^ Furthermore, the practice of pediatrics involves a higher chance of miscalculations and drug prescription errors.^16^

There are different training experiences regarding the communication of bad news.^27,28,29^ However, to our knowledge, training on ME recognition and communication is scarce. Oncology teams have developed successful models for bad-news communication,^30^ which, though not dealing with error communication, help develop or improve communication skills and might work as a foundation to create spaces of similar, error-focused training in our area.

This study had several limitations, which require a careful assessment of results. First of all, it is necessary to clarify that training in the communication of bad news in our hospital is scarce and training programs do not consider the use of simulation tools for this purpose. Secondly, we could only assess 11 groups of resident physicians, so the strength of our results is limited. For one, participants were a sample of residents who might not be representative of all the resident students and, given the fact that they belong to one single care center, the study’s external validity might be affected. In addition, in the actual practice, the health staff who intervenes in cases such as this is made up of attending physicians, nurses and resident physicians. In our training exercise, there were only groups made up of resident physicians, so in real life the situation might work out differently due to the different level of expertise of the medical team (i.e., there may be health personnel who have communication training outside the residency program). However, as referring teachers, we consider that residency is the ideal training opportunity to develop these skills. We also understand that practice within the institution will eventually become more uniform with the implementation of this teaching system.

Furthermore, although the observation form included items which generally required subjective assessment, the three researchers evaluated the performance of each group independently and reached a consensus when there was disagreement. As a strength of this study, it is important that, at the time it was conducted, there were no publications addressing the process of ME communication using a simulation setting. We believe our experience may encourage the use of this type of training aimed at acquiring communication skills with the potential of accelerating the learning processes of the health staff in complex high-fidelity scenarios.

In conclusion, we observed a high number of groups of resident physicians who recognized the presence of a ME in a simulation scenario. However, communication of the error to the family member was proportionally and substantially low, with a predominant concealment of the situation. The communication skills evaluated through the observation form were deficient, according to our observations. Finally, the self-perception of error management by residents was regular and did not change significantly after a debriefing process focused on communicative aspects. We consider it necessary to continue encouraging communication training to enhance medical education and the patient-physician relationship.

## Data Availability

The database that originated the article is available with the corresponding author.
